# Reperfusion Promotes Mitochondrial Dysfunction following Focal Cerebral Ischemia in Rats

**DOI:** 10.1371/journal.pone.0046498

**Published:** 2012-09-28

**Authors:** Jun Li, Xuesong Ma, Wei Yu, Zhangqun Lou, Dunlan Mu, Ying Wang, Baozhong Shen, Sihua Qi

**Affiliations:** 1 Department of Anesthesiology, the Fourth Affiliated Hospital, Harbin Medical University, Harbin, China; 2 Molecular Imaging Key Laboratory of General Universities and Colleges of Heilongjiang Province, Harbin, China; Albany Medical College, United States of America

## Abstract

**Background and Purpose:**

Mitochondrial dysfunction has been implicated in the cell death observed after cerebral ischemia, and several mechanisms for this dysfunction have been proposed. Reperfusion after transient cerebral ischemia may cause continued and even more severe damage to the brain. Many lines of evidence have shown that mitochondria suffer severe damage in response to ischemic injury. The purpose of this study was to observe the features of mitochondrial dysfunction in isolated mitochondria during the reperfusion period following focal cerebral ischemia.

**Methods:**

Male Wistar rats were subjected to focal cerebral ischemia. Mitochondria were isolated using Percoll density gradient centrifugation. The isolated mitochondria were fixed for electron microscopic examination; calcium-induced mitochondrial swelling was quantified using spectrophotometry. Cyclophilin D was detected by Western blotting. Fluorescent probes were used to selectively stain mitochondria to measure their membrane potential and to measure reactive oxidative species production using flow cytometric analysis.

**Results:**

Signs of damage were observed in the mitochondrial morphology after exposure to reperfusion. The mitochondrial swelling induced by Ca^2+^ increased gradually with the increasing calcium concentration, and this tendency was exacerbated as the reperfusion time was extended. Cyclophilin D protein expression peaked after 24 hours of reperfusion. The mitochondrial membrane potential was decreased significantly during the reperfusion period, with the greatest decrease observed after 24 hours of reperfusion. The surge in mitochondrial reactive oxidative species occurred after 2 hours of reperfusion and was maintained at a high level during the reperfusion period.

**Conclusions:**

Reperfusion following focal cerebral ischemia induced significant mitochondrial morphological damage and Ca^2+^-induced mitochondrial swelling. The mechanism of this swelling may be mediated by the upregulation of the Cyclophilin D protein, the destruction of the mitochondrial membrane potential and the generation of excessive reactive oxidative species.

## Introduction

There have been many studies examining the mechanisms involved in ischemic brain injury and reperfusion. Reperfusion is the recirculation of blood flow following transient focal or global ischemia, which is believed to contribute to delayed secondary brain damage [1], [2], [3], [4]. Many lines of evidence have shown that mitochondria, due to playing essential roles in energy metabolism, the generation of reactive oxidative species (ROS), and regulation of the cell death pathway, suffer severe damage in response to ischemic injury [5], [6], [7]. Early classic ultrastructural studies on ischemic neurons concluded that the mitochondria of those neurons were damaged and showed varying degrees of swelling [8], [9], [10]. Current data suggest that mitochondrial swelling may be the result of membrane permeability transition (MPT), which is potentially one of the most important mechanisms of mitochondrial dysfunction following cerebral ischemia, and the occurrence of MPT leading to either necrotic or apoptotic cell death is currently being intensely investigated [11], [12]. Mitochondria assist in maintaining Ca^2+^ homeostasis by sequestering and releasing Ca^2+^. When the mitochondria become overloaded with Ca^2+^, they undergo a cataclysmic MPT. The activation of the MPT by calcium has been investigated to monitor the associated mitochondrial swelling [13], [14], [15]. The MPT is thought to occur after the opening of a channel complex, which has been termed the mitochondrial permeability transition pore (mPTP). Cyclophilin D (Cyp-D) is a mitochondrial member of this channel complex that facilitates the formation of the mPTP [16], [17]. A recent study focused on the mPTP has suggested that the high levels of Cyp-D in neuronal mitochondria result in their greater vulnerability to MPT [18]. The evidence for Cyp-D having an integral role in cerebral ischemic injury originates from experiments performed in Cyp-D-deficient mice, which displayed dramatically reduced brain infarct sizes after ischemic injury compared with wild type mice [19].

The destruction of the mitochondrial energy metabolism is the most immediate cause of mitochondrial dysfunction in cerebral ischemia that is induced as a direct consequence of the impaired delivery of glucose and oxygen to the tissue [20], [21]. The maintenance of the mitochondrial membrane potential (MMP), which established a proton gradient across the inner mitochondrial membrane to provide the driving force that actuates the ATP-synthase to generate high-energy phosphate, was disturbed during cerebral ischemia. The common view is that substantial MMP loss may be a common feature of ischemic injurious processes because these processes favor the opening of the mPTP and the initiation of the apoptotic cascade [22], [23]. Moreover, mitochondria are both targets and sources of oxidative stress, and an excess of ROS has been implicated in the pathogenesis of ischemic cerebral disease. These oxygen radicals are critical contributors to delayed, necrotic neuronal death and powerful initiators of ischemic neural cellular apoptosis [24], [25]. In addition, this highly oxidative stress may damage the mitochondria themselves [26]. More importantly, evidence indicates that mitochondrial ROS production plays a main role in reperfusion injury following cerebral ischemia and that the mechanism by which oxidative stress promotes ischemic neuronal death is related to the occurrence of MPT [27], [28], [29].

Despite the histopathological evidence for reperfusion injury in the form of severe brain damage in stroke models [2], [3], [5], little attention has been focused on the damage to neural cellular mitochondria during the reperfusion process. To address this deficiency, in the current study, mitochondria were isolated at different time points during the reperfusion period following 2 hours of middle cerebral artery occlusion (MCAO). These mitochondria were then analyzed *in vitro* for ultrastructural features and calcium-induced swelling. The expression of Cyp-D, the mitochondrial membrane potential, and the ROS production were then determined to detect mitochondrial dysfunction.

## Materials and Methods

### Materials

Sucrose, EGTA, EDTA, Tris base, Tris-HCl, bovine serum albumin (BSA), calcium chloride, KH_2_PO_4_ [Pi(K)], MgCl_2_, dimethylsulfoxide (DMSO), disodium succinate, 3-[N-Morpholino] propanesulfonic acid (MOPS), and other buffer chemicals were all purchased from Sigma-Aldrich (Beijing, China). Percoll solution was purchased from GE Healthcare (Beijing, China). A bicinchoninic acid (BCA) protein assay kit, polyvinylidene difluoride membranes and all materials for Western blotting were purchased from Thermo Scientific Pierce (Rockford, IL, USA). Cyp-D antibody was purchased from Abcam (Cambridge, MA, USA), cytochrome c oxidase IV (COX IV) antibody was purchased from Santa Cruz Biotechnology (Beijing, China), and horseradish peroxidase-conjugated secondary antibody for Cyp-D and COX IV were obtained from Santa Cruz Biotechnology (Beijing, China). The mitochondrial probes acridine orange 10-nonyl bromide (NAO), tetramethylrhodamine methyl ester (TMRM), and 2′,7′-dichlorodihydrofluorescein diacetate (H_2_DCFDA) were purchased from Invitrogen (Beijing, China). NAO and TMRM were diluted in DMSO and stored in aliquots at 4°C (NAO) or −20°C (TMRM). H_2_DCFDA was diluted in absolute ethanol and stored in aliquots at −20°C. Fresh aliquots were used for each set of measurements.

### Animal Preparation and Experimental Groups

All of the animal protocols used in this study were approved by the Committee on the Guidelines for Animal Experiments of Harbin Medical University, and all of the rats were handled according to the National Institutes of Health Guidelines for the Care and Use of Laboratory Animals. Adult male Wistar rats weighing 250–300 g were divided randomly into 5 groups (total n = 126): ischemia (I) 2 hour group, ischemia/reperfusion (I/R) 2 hour group, I/R 24 hour group and I/R 72 hour group. The I 2 h group was treated with only 2 hours of MCAO (n = 26). Each I/R group underwent 2 hours of MCAO that was followed by 2, 24 or 72 hours of reperfusion, respectively (n = 26 in each group). In addition, a sham-operated group (n = 22) was used as a control.

### MCAO Model

MCAO was conducted as previously described with minor modifications [30], [31] . Briefly, all of the rats were fasted for 8 to 12 hours, with water provided *ad libitum*. The rats were anesthetized using chloral hydrate (350 mg/kg IP); a femoral arterial catheter was inserted; and the blood pressure was continuously monitored. The left common carotid artery, internal carotid artery and external carotid artery were then exposed through a midline incision in the neck, and a monofilament nylon suture (external diameter 0.28 mm) with a silicone-coated tip was inserted into the internal carotid artery approximately 16 to 18 mm from the bifurcation through the external carotid artery stump and gently advanced to occlude the middle cerebral artery. After 2 hours of MCAO, the suture was removed to restore blood flow (reperfusion was confirmed by laser Doppler). During the operative procedures, the body temperature was monitored continuously with a rectal probe and was maintained at 37.0°C±0.5°C with a heating pad and lamp. The mean arterial blood pressure, pH and arterial blood gases were measured before the suture was inserted, at 30 minutes MCAO, and at the beginning of 15 minutes of reperfusion. The sham-operated rats underwent exposure of the vessels without occlusion of the middle cerebral artery. At the beginning of the reperfusion, the rats were tested for neurological deficits by a blinded observer according to a previously described scoring system: 0, no neurological deficits; 1, failure to fully extend right forepaw; 2, circling to the right; 3, falling to the right; and 4, could not walk spontaneously and exhibited depressed levels of consciousness. In the experiments, we only used rats presenting values of 2 or 3.

### Histological analysis

Cerebral damage was assessed by the histological examination of brain sections at the level of the coronal cortex from the sham group, I 2 h group and each I/R group. The animals were deeply anesthetized with chloral hydrate, and subsequently transcardially perfused with 200 mL heparinized 0.9% saline followed by 500 mL 4% paraformaldehyde in 0.1 mol/L phosphate-buffered saline (pH 7.4). The rats were decapitated, and the brains were immersed in 4% paraformaldehyde for 3 d, processed for paraffin embedding, and sectioned (5 µm thick) on a rotary microtome. Coronal sections consisting of the cortex were selected and processed for hematoxylin-and-eosin (HE) staining.

### Isolation of Mitochondria

**Figure 1:**
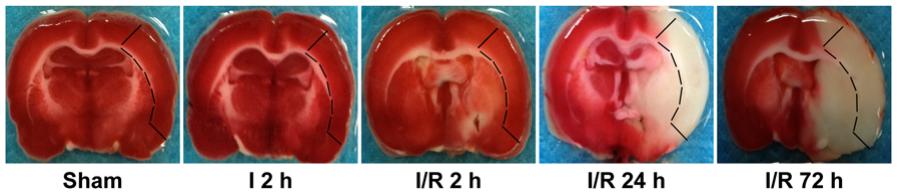
Representative TTC-stained brain sections at 2 h of MCAO, and at 2, 24, and 72 h following 2 h of MCAO or sham MCAO brains. The marked area representes the cortical area separated to isolate mitochondria.

Isolation of rat brain mitochondria was achieved by discontinuous Percoll gradient centrifugation according to a slightly modified method (B) described in a previous study [32]. The animals were decapitated, and the cortical focal tissue was identified as a previous related study [5] and rapidly separated from regions in our animal samples (Figure 1), then dissected on ice and stored in ice-cold isolation buffer (IB) containing 320 mmol/L sucrose, 1 mmol/L EGTA, and 10 mmol/L Tris-Base, pH 7.4. Approximately 500 mg of tissue was manually homogenized in 12% Percoll in IB (10% wt/vol) using a 2 ml Kontes Teflon homogenizer; the homogenate was layered on a gradient of 26% and 40% Percoll in IB and centrifuged at 30,700 *g* for 10 minutes in a Beckman ultracentrifuge (Optima L-90K, Beckman Coulter), yielding a dense fraction in the interface between the 26% and 40% Percoll layers. The resulting dense layer was collected and transferred to a clean tube and diluted 1∶4 in IB. The sample was then washed and centrifuged at 16,700 *g* for 12 minutes. A final washing step using IB and BSA (1 mg/ml) was performed in an Eppendorf microcentrifuge (7,300 *g* for 5 minutes). The mitochondrial pellet was resuspended in IB, and the protein content was determined using the BCA protein assay kit. All of the isolation procedures were performed under ice-cold conditions, and the mitochondria were used within 5 hours of animal decapitation.

### Electron Microscopy

Ultrastructural changes in isolated brain mitochondria were assessed by transmission electron microscopy using the method described below. An aliquot of the final mitochondrial suspension from the sham group, I 2 h group and each I/R group was re-centrifuged at 10,000 *g* for 5 minutes. The mitochondrial pellet was fixed with 4% glutaraldehyde in 0.1 mol/L cacodylate buffer (pH 7.4) and postfixed with 1% osmium tetroxide. The preparations were dehydrated through an ethanol gradient, processed for Epon 812 embedding, and sectioned at a thickness of 80 nm on a rotary microtome. The ultrathin sections were stained with 4% uranyl acetate-lead citrate and examined with a Tecnai G2 Transmission Electron Microscopy (FEI, USA).

### Measurement of Mitochondrial Swelling

An increased mitochondrial permeability transition was recognized as a swelling of isolated mitochondria induced by adding Ca^2+^ and was assayed spectrophotometrically as previously described [33], [34]. The mitochondrial pellet isolated after the last washing step was resuspended in ice-cold BSA-free and EDTA-free sucrose buffer (300 mmol sucrose and 10 mmol/L Tris-Base, pH 7.4), and an aliquot of mitochondria (100 µg) was finally diluted in 1 ml BSA-free and EDTA-free sucrose buffer at 25°C. Swelling was initiated by the addition of 100–1000 µmol/L CaCl_2_ and evaluated as a decrease in light scattering transmission at 540 nm for 5 minutes with a spectrophotometer (UNICO 2100UV, China).

### Western Blot Analysis

Western blot analysis was performed to measure the expression of the Cyp-D protein in all of the groups. Mitochondrial suspensions were lysed on ice in 100 µl RIPA buffer (50 mmol/L Tris-HCl (pH 7.4), 150 mmol/L NaCl, 1 mmol/L PMSF, 1 mmol/L EDTA, 1% Triton X-100, 0.5% sodium deoxycholate, and 0.1% SDS). The lysates were cleared by centrifugation at 12,000 *g* for 10 minutes, and the pellet was resuspended in IB. Then, 100 µg of mitochondrial protein normalized using the BCA method was denatured in SDS gel-loading buffer (100 mmol/L Tris-HCl, 200 mmol/L dithiothreitol, 4% SDS, 0.2% bromophenol blue, and 20% glycerol) at 95°C for 5 minutes and separated on 12% SDS-polyacrylamide gels. After electrophoresis, the protein obtained from each sample was transferred to polyvinylidene difluoride membranes. The blots were rinsed with Tris-buffered saline (TBS) containing 0.1% Tween (TBST) and then blocked with 5% skim milk for 1 hour. The membranes were probed with a primary antibody against Cyp-D (Catalog NO. ab3567) at a concentration of 1∶1000 overnight at 4°C, and with a primary antibody against COX IV (Catalog NO. sc-69360) blotted as controls at a concentration of 1∶1000 overnight at 4°C. These two antibodies recognized the 37 kDa Cyp-D protein and the 17 kDa COX IV. After the blots were washed two times in TBST for 10 minutes and once in TBS for 10 minutes, the membranes were incubated with a horseradish peroxidase-conjugated secondary antibody for Cyp-D (1∶2000) and COX IV (1∶2000); the protein bands were visualized with ECL; and the immunoreactivity signals were quantified by densitometry. The relative changes in the Cyp-D levels were represented as the ratio of the optical density value between Cyp-D and COX IV.

### Flow Cytometry Analysis of Mitochondrial Membrane Potential and Mitochondrial ROS Production

Flow cytometric analysis was performed using a FACSCalibur equipped with a 488 nm argon laser and a 635 nm red diode laser (BD, USA). The data from the experiments were analyzed using the CellQuest software (BD, USA). The geometric mean fluorescence intensities were calculated from logarithmically amplified signals, and the results are reported as linear values. To exclude debris, samples were gated based on light scattering properties in the side scattering (SSC) and forward scattering (FSC) modes, and 10000 events per sample within the R1 gate were collected. For staining, 50 µg of the mitochondrial samples for each group was suspended in 300 µl of analysis buffer (250 mmol/L sucrose, 20 mmol/L MOPS, 10 mmol/L Tris-Base, 100 umol/L Pi(K), and 0.5 mmol/L Mg^2+^, pH 7.0) containing 5 mmol/L succinate either with or without probe. To check the purity of the analyzed gate, each mitochondrial sample was selectively stained with NAO (100 nmol/L, excitation at 488 nm and emission at 525 nm), which binds to cardiolipin in the inner mitochondrial membrane. The NAO-positive mitochondria were then stained with TMRM (100 nmol/L, excitation at 488 nm and emission at 590 nm) and H_2_DCFDA (10 µmol/L, excitation at 488 nm and emission at 525 nm), which were used to measure the mitochondrial membrane potential and the production of ROS. All of the samples were incubated at room temperature and protected from light during the 10-minute incubation with NAO and TMRM and the 30-minute incubation with H_2_DCFDA. The autofluorescence (geometric mean value; CellQuest) from the control samples (no probe) was subtracted from that of the stained samples before comparison. The mitochondrial NAO signal, membrane potential and ROS generation were analyzed by FACScan for changes in FL2-H of TMRM and FL1-H of NAO and H_2_DCFDA.

### Statistical Analysis

The quantitative data were expressed as the means±standard deviation and analyzed using a one-way analysis of variance (ANOVA) and the Bonferroni *post hoc* test. P<0.05 was considered statistically significant.

## Results

### Physiological Data

The physiological parameters of mean arterial blood pressure, pH, core temperature, and arterial blood gas tension were monitored and maintained within normal ranges for all of the experimental animals (**Table 1**). No neurological deficits were observed in the sham group rats, whereas all of the neurological assessment values in the rats subjected to MCAO were rated as 2 or 3 in each of the experimental animals.

**Table 1:** Physiological parameters.

Parameters	Time	Group				
		Sham	I 2 h	I/R 2 h	I/R 24 h	I/R 72 h
MAP (mm Hg)	Baseline	93±5	88±4	92±6	93±4	92±6
	Ischemia	90±3	92±4	89±2	92±3	91±3
	Reperfusion	91±3	92±4	93±4	93±2	89±6
PaO_2_ (mm Hg)	Baseline	104±6	101±7	99±5	101±4	103±8
	Ischemia	98±7	102±5	101±6	105±5	103±7
	Reperfusion	100±6	102±5	98±5	102±3	101±6
PaCO_2_ (mm Hg)	Baseline	35±2	36±3	36±3	34±5	35±4
	Ischemia	36±5	37±4	36±3	36±2	36±5
	Reperfusion	36±4	36±5	37±4	35±4	35±3
pH	Baseline	7.37±0.04	7.39±0.03	7.38±0.03	7.37±0.02	7.38±0.04
	Ischemia	7.40±0.02	7.41±0.03	7.40±0.03	7.39±0.03	7.38±0.03
	Reperfusion	7.39±0.03	7.41±0.03	7.41±0.04	7.38±0.03	7.37±0.04
Rectal temperature	Baseline	36.9±0.3	36.9±0.3	36.5±0.4	36.6±0.3	36.8±0.3
	Ischemia	36.6±0.5	36.7±0.3	36.2±0.3	36.5±0.5	36.7±0.3
	Reperfusion	36.4±0.3	36.5±0.2	36.7±0.4	36.4±0.3	36.6±0.2

All values are mean ± SD. Arterial blood gas tensions include PaO_2_, PaCO_2_ and pH.

MAP, mean arterial pressure; PaO_2_, arterial oxygen pressure; PaCO_2_, arterial carbon dioxide pressure.

### Histological Changes after Focal Cerebral Ischemia-Reperfusion

**Figure 2:**
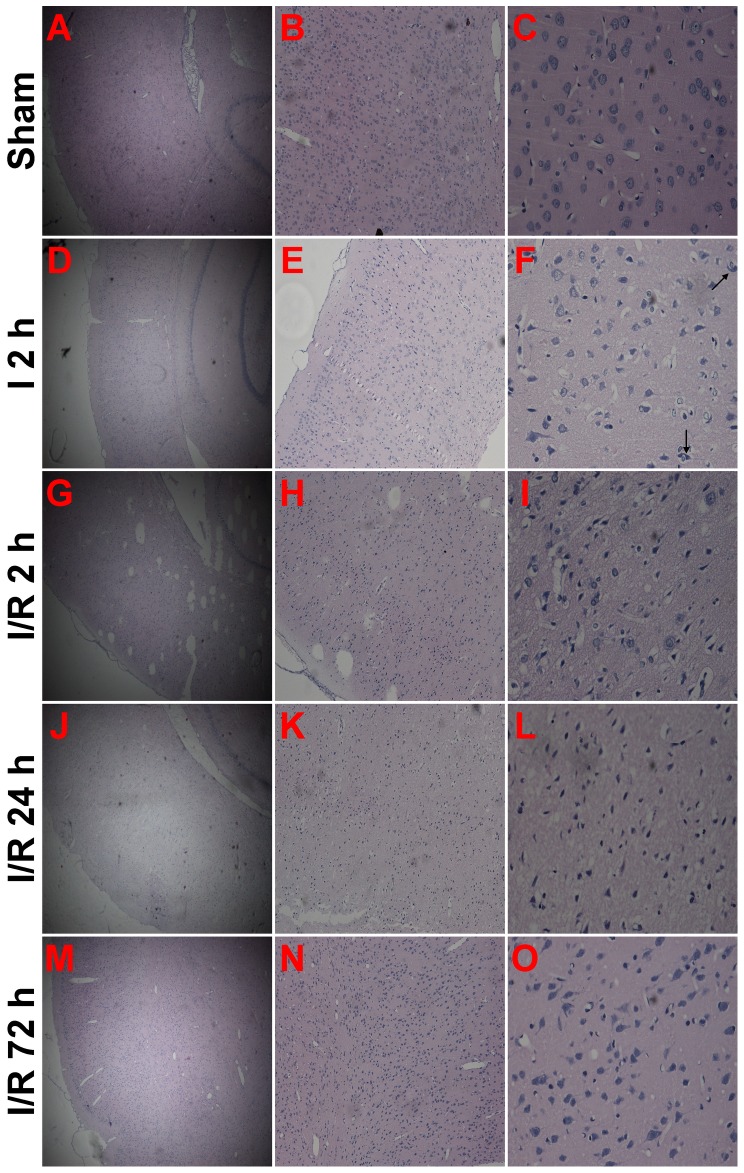
Representative histological characteristics of brain sections at the level of the cortex were assessed by hematoxylin-and-eosin (HE) staining examination. (**A–C**) Sham cortical sections revealed normal cortical tissue (n = 2). (**D–F**) Cortical sections from the I 2 h group showed cortical tissue injury (n = 4). Damaged neurons (arrowheads) were surrounded by activated glia cells. (**G–I**) Cortical sections from the I/R 2 h group showed aggravated cortical tissue injury (n = 4). (**J–L**) Cortical sections from the I/R 24 h group showed a nearly completely damaged nerve cells (n = 4). (**M–O**) Cortical sections from the I/R 72 h group showed improved residual neurons in the damaged cortex and more activated glial cells (n = 4). Scale bars: A, D, G, J and M, 50 µm; B, E, H, K and N, 125 µm; C, F, I, L and O, 500 µm.

There were no morphological injuries to the cortical neurons in the sham group, as these neurons displayed normal integrity of their cell structure (**Figures 2A–C**). After 2 hours of MCAO, the neurons began to appear damaged, as shown by the disappearance of the nuclear structure and the appearance of cytoplasmic eosinophilia. The hyperchromatic nuclei of activated glial cells appeared round or elongated (**Figures 2D–F**). Compared with the I 2 h group, more damaged neurons were evident in the group that experienced reperfusion for 2 hours after 2 hours of MCAO. In this group, the extent of damage was increased, and activated glial cells were also visible (**Figures 2G–I**). At 24 hours after ischemia, almost all of the neurons were damaged. The increasing pallor of perikarya made the identification of neurons difficult, and the remnants of pyknotic nuclei could only be observed in the cortical area (**Figures 2J–L**). In contrast to the I/R 24 h group, the structure of residual neurons was improved, as evidenced by visible membranes and nuclei, as well as more glial cells in the cortical area. (**Figures 2 M–O**).

### Ultrastructural Changes in Isolated Mitochondria

**Figure 3:**
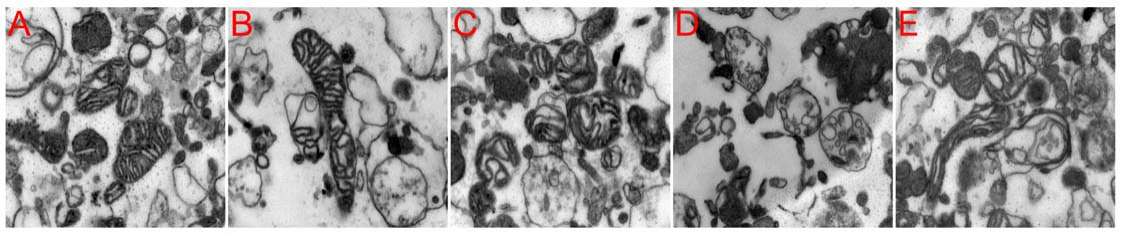
Representative isolated mitochondrial ultrastructural changes as assessed by electron microscopy. (**A**) Sham group (n = 2). (**B**) Mitochondria isolated from 2 hours middle cerebral artery occlusion (MCAO) (n = 4). (**C–E**) Mitochondria isolated from groups exposed to 2, 24 and 72 hours of reperfusion following 2 hours of MCAO (n = 4 each group)(magnification 26500×).

The mitochondria in the sham group appeared to exhibit normal membrane integrity. The cristae showed no signs of swelling or injury, and a typical homogeneous staining pattern of the matrix was observed, without density changes (**Figure 3A**). After 2 hours of MCAO, the mitochondria showed swelling with poorly defined cristae and decreased matrix density (**Figure 3B**). Similarly to the mitochondria of the I 2 h group, the mitochondria in the group that experienced 2 hours of reperfusion following 2 hours of MCAO were moderately to severely swollen, the inner and outer membranes were disrupted, and the intracristal space was dilated (**Figure 3C**). In contrast to the I/R 2 hour group, the I/R 24 hour group exhibited a more severe collapse of the mitochondrial membrane structures in both the inner and outer membranes. Almost all of the mitochondria lost their typical rounded or tubular morphology and exhibited an irregular shape; in addition, the matrix was electron-lucent with fragmented cristae, and extreme dilation of the intracristal space was observed (**Figure 3D**). With respect to the extension of the reperfusion time, the mitochondria in the I/R 72 hour group showed less severe morphological changes compared with the I/R 24 hour group, certain of these mitochondria were still swollen and exhibited collapsed membranes, whereas others of them appeared to have less damaged inner and outer membranes. The mitochondria in this group also presented decreased matrix density with moderately disrupted cristae and dilated intracristal spaces (**Figure 3E**).

### Changes in Mitochondrial Swelling Induced by Ca^2+^

**Figure 4:**
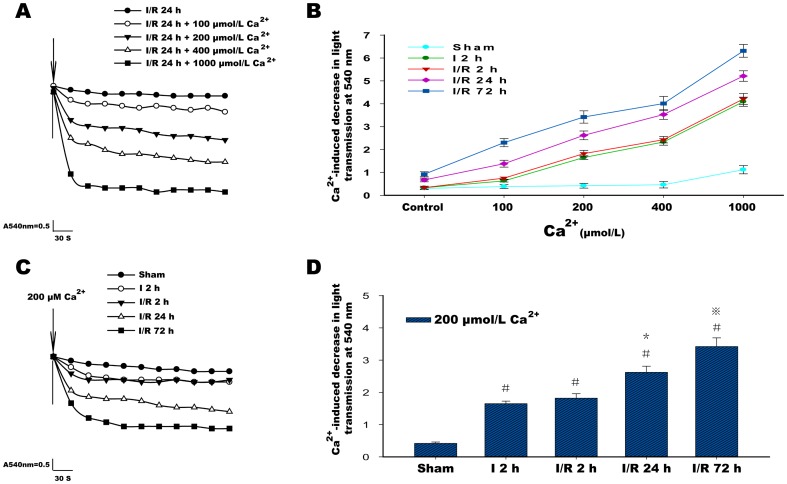
Ca^2+^ induced swelling in isolated mitochondria. (**A**) Representative traces of 100–1000 µmol/L Ca^2+^ induced a decrease in light transmission in the ischemia/reperfusion (I/R) 24 h group. (**B**) Graph showing that the Ca^2+^-induced reduction in light transmission was dose dependent at concentrations of 100–1000 µmol/L (compared with control or compared with each other between the different concentrations of calcium; in each group, there was a statistically significant difference). This reduction gradually increased with the extension of reperfusion time from 2 hours to 72 hours (from 100 to 1000 µmol/L Ca^2+^, compared with each other between the three reperfusion groups, there was a statistically significant difference). The control in B indicates without Ca^2+^ intervention. (**C**) Representative traces of 200 µmol/L Ca^2+^ induced a decrease in light transmission in the sham group, the ischemia (I) 2 h group and the ischemia/reperfusion (I/R) 2 h, I/R 24 h and I/R 72 h groups. (**D**) A graph showing that the 200 µmol/L Ca^2+^-induced reduction in light transmission was increased with the extension of the reperfusion time from 2 hours to 72 hours. Six animals were included in each group. Each bar represents the means±SD. ^#^P<0.05 versus sham; ^*^P<0.05 versus I 2 h, I/R 2 h and sham; ^⋇^P<0.05 versus I 2 h, I/R 2 h, I/R 24 h and sham.

There was no significant decrease in the mitochondrial transmission values in isolated mitochondria without Ca^2+^ intervention in each group. In the sham group, the mitochondrial transmission values showed a mild decrease after 5 minutes of exposure to increased Ca^2**+**^ concentrations and a significant reduction at 1000 µmol/L Ca^2+^. In the I 2 h group, the mitochondria began to respond to Ca^2+^ intervention with a more severe decrease in light transmission, especially 400 and 1000 µmol/L Ca^2+^. However, in isolated mitochondria from each reperfusion group, it was clear that exposure to Ca^2+^ induced a significant and dose-dependent decrease in light transmission, and this trend was increased as the reperfusion time was extended and was particularly evident at 72 hours of reperfusion (**Figures 4A and B**). Because of the significant and stable decrease in light transmission induced by treatment with 200 µmol/L Ca^2+^, relative changes in light transmission for isolated mitochondria in I 2 h group and the 2 to 72 hour reperfusion groups were shown with 200 µmol/L Ca^2+^ treatment (**Figures 4C and D**).

### Cyp-D Expression in Isolated Mitochondria

**Figure 5:**
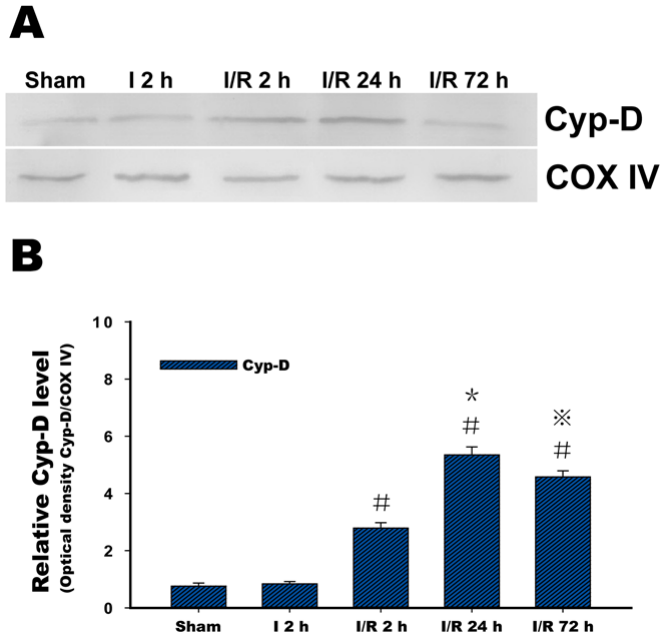
Representative cyclophilin D (Cyp-D) protein expression. (**A**) Western blots showing representative Cyp-D protein expression. COX IV was blotted as a control and indicated no changes during the time course of the experiment. (**B**) Relative analysis of the Cyp-D protein levels in isolated mitochondria in the sham group, the ischemia (I) 2 h group, and the ischemia/reperfusion (I/R) 2 h, I/R 24 h and I/R 72 h groups. Six animals were included in each group. Each bar in B represents the means±SD. ^#^P<0.05 versus I 2 h and sham; ^*^P<0.05 versus I 2 h, I/R 2 h and sham; ^⋇^P<0.05 versus I 2 h, I/R 2 h, I/R 24 h and sham.

There was a very low level of Cyp-D protein expression in the isolated mitochondria in the sham group. In the I 2 h group, Cyp-D protein was not induced to express significantly by the 2 hours of MCAO. However, in contrast to the sham and I 2 h group, Cyp-D protein expression began to increase in isolated mitochondria after 2 hours of reperfusion and continued to increase, peaking at 24 hours of reperfusion, whereas at 72 hours, these values appeared to decrease (**Figure 5**).

### Analysis of the Light Scattering Properties of Mitochondria

**Figure 6:**
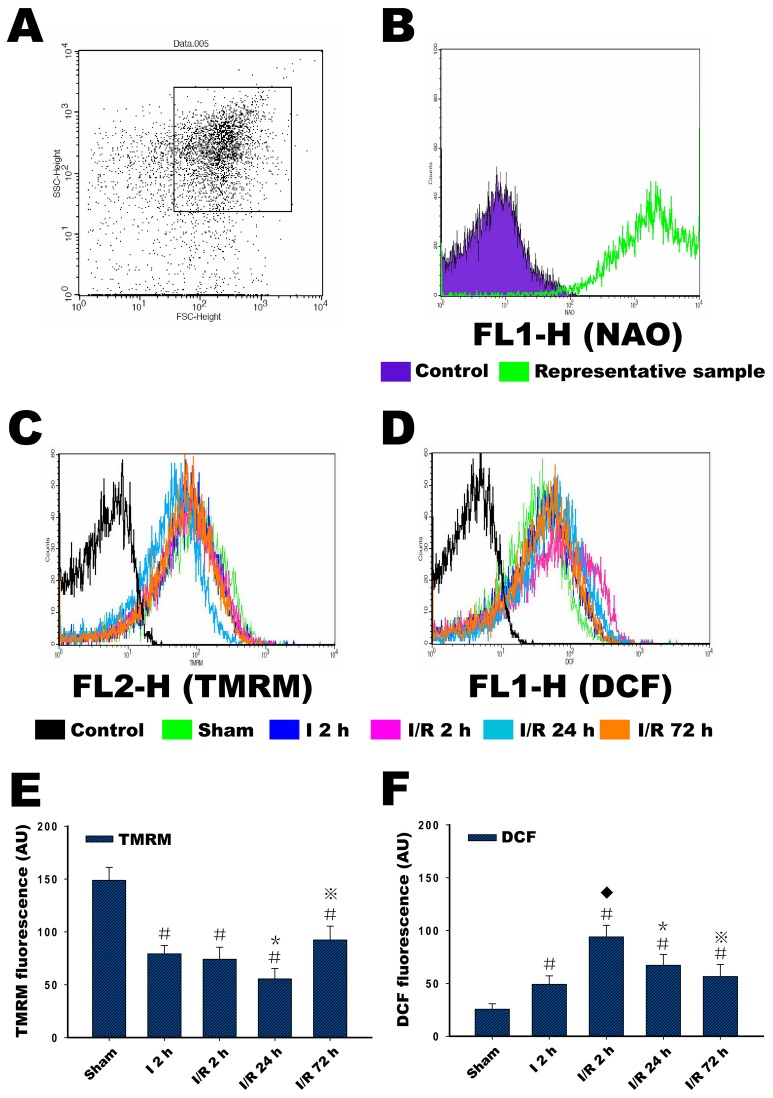
Selection of mitochondria based on their light scattering properties and NAO staining, and measurement of membrane potential and ROS production in isolated mitochondria. (**A**) In the FSC/SSC plot of the isolated mitochondria, 10000 events were collected within gate R1. (**B**) To assess the purity of mitochondria, the preparations were analyzed for NAO fluorescence, and almost all of the events in gate R1 were positive for NAO (M2) compared with samples without staining (M1), which confirmed that they were mitochondria. (**C**) Changes in the membrane potential in isolated mitochondria in the sham group, in the ischemia (I) 2 h, and in the ischemia/reperfusion (I/R) 2 h, I/R 24 h and I/R 72 h groups, as reflected by changes in TMRM fluorescence intensity. (**D**) Changes in ROS production in isolated mitochondria in the sham group, the ischemia (I) 2 h, and in the I/R 2 h, I/R 24 h and I/R 72 h groups, reflecting the changes in DCF fluorescence intensity. (**E and F**) The relative changes in TMRM fluorescence intensity and DCF fluorescence intensity. Six animals were included in each group. Each bar represents the means±SD. ^#^P<0.05 versus sham; ^⧫^P<0.05 versus I 2 h; ^*^P<0.05 versus I 2 h, I/R 2 h and sham; ^⋇^P<0.05 versus I 2 h, I/R 2 h, I/R 24 h and sham. Control in **C** and **D** indicates samples not stained with probes.

The purity of the mitochondrial preparations was determined by staining with NAO. In the FSC/SSC plot of the isolated mitochondria, 10000 events were collected within gate R1, which we assumed to be the mitochondrial fraction (**Figure 6A**). When the events within R1 were plotted with respect to the fluorescence at 525 nm for the NAO-stained samples, almost all of the events in gate R1 were positive for NAO (M2) compared with samples without staining (M1), which confirmed that they were mitochondria (**Figure 6B**). Thus, the gated R1 events were subsequently used for staining with TMRM and H_2_DCFDA.

### Changes in Mitochondrial Membrane Potential

The mitochondrial sample within the R1 gate was confirmed with NAO staining and then loaded with TMRM to measure the MMP. In the sham group, the mitochondria exhibited a high degree of polarization, as indicated by a high TMRM fluorescence intensity. In contrast to the mitochondria of the sham group, 2 hours of ischemia significantly reduced the TMRM fluorescence intensity, which indicates a decrease in the MMP. After 2 hours of reperfusion, this initial reduction in the TMRM fluorescence intensity continued but was similar in magnitude to that observed in the ischemic group. This reduction was greatest after 24 hours of reperfusion. As the reperfusion time was extended to 72 hours, the TMRM fluorescence intensity was more intense compared with that observed at 24 hours of reperfusion, indicating the increase of the MMP at this time point (**Figures 6C and E**).

### Changes in Mitochondrial ROS Production

Because H_2_DCFDA is oxidized to the fluorescent compound DCF when it reacts with ROS inside mitochondria, the DCF fluorescence intensity actually reflects the ROS generation of mitochondria. Similarly, the mitochondrial sample within the R1 gate (after confirmation with NAO staining) was loaded with H_2_DCFDA. Mitochondria exhibit a basal level of ROS production, leading to a certain DCF fluorescence value in the sham group compared with the control sample. In the I 2 h group, the mitochondria demonstrated a significant increase in ROS production compared with the sample in the sham group. In contrast to the mitochondria of the sham and I 2 h group, ROS production was extremely increased, as indicated by the highest level of DCF fluorescence intensity. As the reperfusion time extended to 72 hours, the ROS production moderately and gradually decreased compared with that observed at 2 hours of reperfusion time. However, the ROS still remained high compared with the sham group (**Figures 6D and F**).

## Discussion

The main finding of the present study is that the characteristic changes involved in isolated mitochondrial dysfunction occur continuously throughout the ischemia-reperfusion period following 2 hours of focal cerebral ischemia in rats. This mitochondrial injury resulting from the ischemia-reperfusion injury led to severe morphological damage to the mitochondria and a significant Ca^2+^-concentration-dependent increase in mitochondrial swelling. Mitochondrial dysfunction was also indicated by measurements of increased mitochondrial Cyp-D protein expression, decreased mitochondrial membrane potential and increased mitochondrial ROS production, all of which could be a part of the mechanism of cerebral ischemic reperfusion injury. Improvements in the methods for mitochondrial isolation have made *in vitro* studies of mitochondria possible. In this study, Percoll density gradient centrifugation was used to isolate non-synaptic mitochondria. Previous studies have shown that the mitochondria produced from this method contain little contamination and maintain high rates of respiratory activity [32], [35]. This mitochondrial fraction originates from neurons and other cell types, including glia [36]. Research indicates that following 90–120 minutes MCAO, irreversibly injured brain tissue can be clearly distinguished from the early onset of reperfusion up to 7 days after reperfusion, especially in the striatum and the cortex [37]. As shown in our TTC-stained brain sections, rats subjected to 2 hours of MCAO following reperfusion showed the development of gradually increased cerebral infarction. Severe cortical infarction appeared at 24 hours after MCAO, and this damage persisted at 72 hours. The same cortical infarct area was identified for mitochondrial isolation in each experimental group, as marked in Figure 1. Cortical tissue in our study, examined by HE staining, demonstrated characteristics of cortical tissue damage caused by the focal cerebral ischemia and reperfusion injury, and these features also confirmed the reperfusion-related infarction reflected by TTC staining.

Among the numerous classic histopathological studies describing mitochondrial changes after ischemia, mitochondrial swelling remains the most universal of ultrastructural changes [10], [38], [39]. In the present study, after 2 hours of MCAO, the mitochondria showed swelling, and this moderate form of injury continued into the reperfusion period. The observed mitochondrial damage reached its peak after 24 hours of reperfusion and induced catastrophic degeneration and loss of membrane shape and form. When the reperfusion period was extended to 72 hours, the mitochondrial damage was alleviated, and less severe swelling and relative integrity of the membrane and crystal structure were observed. Differences in the mitochondrial ultrastructure between ischemic mitochondria and reperfused mitochondria in a slice of the MCAO rat model were found in another study, which also showed that the most severe destruction of mitochondria occurred after 24 hours of reperfusion following 2 hours MCAO. This change was greater than the damage caused by permanent occlusion of the middle cerebral artery, but examination of longer reperfusion times was not performed in this previous study [40]. In addition, the pathological changes observed in nerve cells were similar to the mitochondrial ultrastructural changes. These results indicate that the mild to moderate impairment of mitochondria at the conclusion of 2 hours of MACO and 2 hours of reperfusion may be caused by the earlier, gradually increased, ischemia and reperfusion injury. Additionally, the extremely damaged mitochondria detected after 24 hours of reperfusion may be derived from residual damaged neurons, and the increasing glial cells at 72 hours of reperfusion may contribute to improved mitochondria.

Several studies have found that mitochondria may undergo MPT as a result of calcium accumulation, oxidative stress and other cell death signals that occur as a result of ischemic brain injury [12], [41], [42]. The phenomenon described as MPT is transmitted through the opening of mPTP and leads to the dissipation of the mitochondrial transmembrane potential and an influx of solutes. As a result, swelling of the mitochondria occurs, eventually leading to damage of the mitochondrial membrane. Previous studies have shown that calcium can cause mitochondrial permeability transition and act as an indicator of mitochondrial swelling [13], [15]. Measurement of the swelling induced by calcium in isolated mitochondria after cerebral ischemia-reperfusion was examined in this study; the results demonstrated that there was a dose- dependent decrease in light transmission as the concentration of calcium was increased from 100 to 1000 µmol/L in each group, which indicates the reduced capacity of the mitochondria to regulate calcium homeostasis, especially in the ischemia or reperfusion group. Because significant and stable swelling was induced by 200 µmol/L Ca^2+^, a more notable finding was that the mitochondria in each reperfusion group exhibited a gradual increase in swelling as the reperfusion time was lengthened, with maximum swelling occurring after 72 hours of reperfusion. This effect can be explained by the reduced capacity of mitochondria following ischemia-reperfusion. The features observed in the assessment of Ca^2+^-induced swelling were not consistent with changes in the general morphology of the mitochondria, which may be because the capacity of the mitochondria to respond to the additional intervention was not recovered despite the improvement in the mitochondrial structure after 72 hours of reperfusion.

Given the important role of the mPTP in mitochondrial dysfunction, the structure of the mPTP has been determined using molecular biology techniques; the basic unit of the pore has been proposed to be a complex of the voltage-dependent anion channel (VDAC, porin) in the outer mitochondrial membrane in association with the inner membrane adenine nucleotide translocase (ANT) and the matrix isomerase Cyp-D [16], [17]. A subsequent study found that neuronal mitochondria contain higher levels of Cyp-D, and the high levels of Cyp-D in neuronal mitochondria result in their greater vulnerability to MPT [18]. In a groundbreaking study, Cyp-D gene-knockout mice were exposed to a focal ischemic attack and exhibited significantly smaller infarcts than non-knockout mice, implying that Cyp-D is involved in ischemic cerebral injury [19]. A recent study also found a significant reduction in brain injury when Cyp-D-deficient mice were subjected to cerebral hypoxia-ischemia. In addition, this study provided evidence that Cyp-D is critical for the development of adult brain injury, and the explanation of this phenomenon is that the role of Cyp-D in hypoxia-ischemia shifts from a predominantly prosurvival protein in the immature brain to a cell death mediator in the adult brain [43]. To further understand the role of Cyp-D in cerebral ischemia-reperfusion, we examined Cyp-D protein expression at 2 hours MCAO and during reperfusion. We found that Cyp-D protein was not significantly induced after 2 hours of MCAO, whereas increased Cyp-D protein expression began at 2 hours of reperfusion. Peak expression was obvious at 24 hours, and expression subsequently decreased when the reperfusion was extended to 72 hours. These results demonstrated that in the 2-hour focal cerebral ischemia model, reperfusion accounts for the primary induction of Cyp-D expression. This expression corresponded to changes in mitochondrial ultrastructure, which suggestes a strong relationship between Cyp-D and mitochondrial swelling during ischemia-reperfusion.

The mitochondrion is the primary organelle for the production of high-energy phosphate; additionally, impaired mitochondrial energy metabolism in global and focal cerebral ischemic injury has been reported [20], [21], [44]. Ischemia causes a depletion of the materials necessary to produce this phosphate and strongly affects the electron transport chain. The MMP reflects the performance of the electron transport chain and can indicate a pathological disorder of mitochondrial energy metabolism. Studies *in vivo* have determined that the MMP may dissipate during oxygen-glucose deprivation (OGD) procedures in cultured neurons and re-polarize following reoxygenation. The time course of changes in MMP depends on the duration of the OGD, and these studies also demonstrate that more than 60 minutes of OGD result in depolarization with no repolarization following reoxygenation [45], [46]. Our study showed that ischemia causes a significant decrease in MMP; reperfusion after 2 hours of MCAO may still impair mitochondrial energy regulation, which is consistent with the changes we observed in the characteristics of mitochondrial morphology and the expression of the Cyp-D protein. Our interpretation of this phenomenon is that the continuous low MMP status at the early time of reperfusion may result from the long period of ischemia injury (2 hours of MCAO), and this disruption of the mitochondrial energy supply may be the direct cause of other mitochondrial dysfunctions. Similarly, this inhibition of the mitochondrial energy metabolism during the reperfusion period was insufficient to provide the energy necessary to maintain the survival of nerve cells; therefore, neuronal death occurred at the end of ischemia and early reperfusion. Specifically, extremely decreased MMP at 24 hours of reperfusion corresponded to a status that nerve cells could not survive. The increased MMP at 72 hours of reperfusion may be due to the contribution of mitochondria from activated glial cells.

Mitochondria are the sites of ROS generation, and they function in several defense mechanisms under normal conditions. In our results, mitochondria exhibited a basal level of ROS production, so the relative increase in DCF fluorescence was measured and compared in each group to determine the relative changes in ROS production. Another mechanism thought to cause mitochondrial dysfunction is the production of ROS, which are synthesized in excess during and after ischemia [47]. Both *in vitro* studies with neural cells and *in vivo* animal models of global cerebral ischemia have demonstrated that mitochondrial energy metabolism is extremely sensitive to impairment by reactive oxygen and that mitochondrial oxidative stress both limits metabolic recovery and promotes apoptosis [48], [49]. Increases in the accumulation of ROS have been detected during focal ischemia [50], and a similar result was observed in our experiment, namely, that 2 hours of MCAO induced a moderately increased generation of ROS. More important, our results demonstrated a continuous trend of mitochondrial ROS production during reperfusion for the first time, especially the surge of production at an early time of reperfusion. These results also make it clear that under conditions of mitochondrial damage and a loss of normal metabolic regulation, a large and rapid dose of reoxygenation in the brain may lead to excessive ROS production, suggesting that there is a meaningful association between mitochondrial dysfunction and its ability to regulate ROS production. Reactive oxygen can also induce the release of cytochrome c from mitochondria through the promotion of the MPT, an event that is likely to cause necrosis due to the devastating effects of the MPT on mitochondrial energy metabolism [51], [52]. Given the results observed in this study, we believe that the early increase in mitochondrial ROS production during reperfusion is one of the initial causes of other types of mitochondrial dysfunction, and this ROS production could also be one of the causes of reperfusion-induced cerebral pathological damage.

In conclusion, reperfusion following 2 hours of focal cerebral ischemia induced significant mitochondrial morphological damage and significant Ca^2+^-induced mitochondrial swelling. The mechanism of this swelling may be mediated by the upregulation of the Cyp-D protein, destruction of the mitochondrial membrane potential and excessive ROS generation. The disruption of mitochondrial energy metabolism and high oxidative stress may be the direct and initial causes of this mitochondrial dysfunction.
